# Peptides as Therapeutic Agents for Inflammatory-Related Diseases

**DOI:** 10.3390/ijms19092714

**Published:** 2018-09-11

**Authors:** Sara La Manna, Concetta Di Natale, Daniele Florio, Daniela Marasco

**Affiliations:** Department of Pharmacy, MASBC, Metodologie Analitiche per la Salvaguardia dei Beni Culturali University of Naples “Federico II”, 80134 Naples, Italy; floriodaniele1@gmail.com (D.F.); daniela.marasco@unina.it (D.M.)

**Keywords:** inflammatory diseases, anti-inflammatory peptides, peptides as therapeutic

## Abstract

Inflammation is a physiological mechanism used by organisms to defend themselves against infection, restoring homeostasis in damaged tissues. It represents the starting point of several chronic diseases such as asthma, skin disorders, cancer, cardiovascular syndrome, arthritis, and neurological diseases. An increasing number of studies highlight the over-expression of inflammatory molecules such as oxidants, cytokines, chemokines, matrix metalloproteinases, and transcription factors into damaged tissues. The treatment of inflammatory disorders is usually linked to the use of unspecific small molecule drugs that can cause undesired side effects. Recently, many efforts are directed to develop alternative and more selective anti-inflammatory therapies, several of them imply the use of peptides. Indeed, peptides demonstrated as elected lead compounds toward several targets for their high specificity as well as recent and innovative synthetic strategies. Several endogenous peptides identified during inflammatory responses showed anti-inflammatory activities by inhibiting, reducing, and/or modulating the expression and activity of mediators. This review aims to discuss the potentialities and therapeutic use of peptides as anti-inflammatory agents in the treatment of different inflammation-related diseases and to explore the importance of peptide-based therapies.

## 1. Introduction

**Figure 1:**
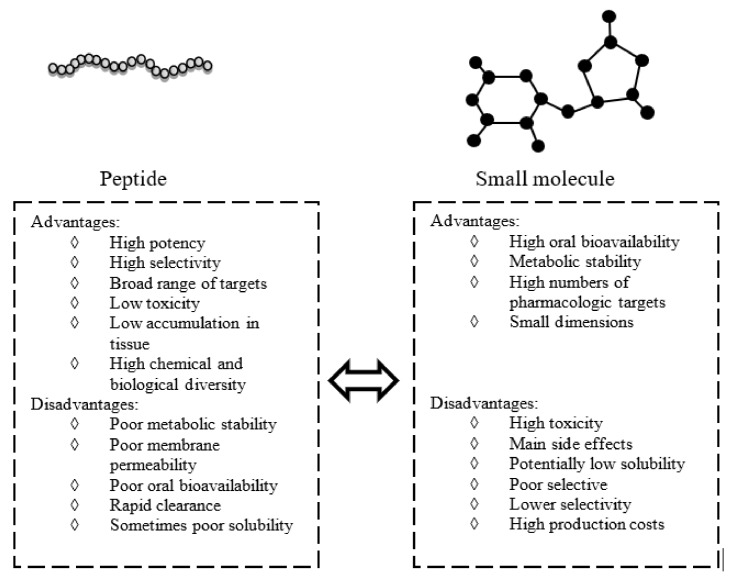
Peptides versus small molecules: advantages and disadvantages.

Inflammation is a natural defensive response of our body to toxic stimuli, such as injury and infection, that could lead to damage of involved tissues [1]. The inflammatory response includes the activation of different biological mechanisms like the production and secretion of pro-inflammatory mediators such as cytokines, chemokines, interleukins, and growth factors [2]. The regulation of the inflammatory process is essential to maintain or restore homeostasis in damaged biological compartments, and alteration of this regulation is associated with different human diseases [1,3,4]. The link between inflammation and cancer is widely accepted: the prolonged presence of inflammatory cells in the tumor environment often accelerates its growth; indeed, inflammatory cells and mediators are constituents of tumor microenvironment [5,6]. The treatment of inflammatory and cancer disorders often implies the use of small molecule as drugs able to interact with a great number of pharmacological targets. Unfortunately, high toxicity, low selectivity, and a wide range of undesired side-effects are associated with them, including brain–blood barrier cross and the generation of toxic molecules after metabolic process; they are often inadequate in long therapy [7] (Figure 1). An alternative treatment, based on the use of bioactive peptides as anti-inflammatory agents, is being developed [8].

As schematically reported in Figure 1, peptides, with respect to small molecules, usually possess higher potency and selectivity for their targets, mainly because of their chemical and consequent biological diversity. Several natural therapeutic peptides, such as insulin, demonstrated low toxicity and fast clearance [9], and others show good membrane penetration. Thus, the amount required to obtain a therapeutic effect is lower when compared with small molecules [10]. The yield of peptide synthesis is highly sequence dependent and, when the complexity of the process increases, analytical characterization is critical, leading to a great increase in associated costs and time required. Moreover, unless they are chemical modifications, they present poor metabolic stability and oral bioavailability, and can only be injected [11]. Recent efforts in chemical modification reactions, new formulations, new drug delivery systems, and novel administration routes have overcome these disadvantages, making them good starting points to develop novel anti-inflammatory drugs. All these factors prompt to a recent renaissance of peptides as therapeutic compounds and make them competitive on the pharmaceutical market [12].

Several endogenous peptides secreted during inflammatory response, as well as others deriving from interacting regions of protein complexes triggered by inflammatory mediators, showed anti-inflammatory properties and might be engaged in the development of new therapies [9,10].

## 2. Peptides and Peptidomimetics as Modulators of Inflammation

This review aspires to discuss examples of peptides as anti-inflammatory compounds and their employment in therapies, the sequences of reported cases are shown in Table 1.

Cell life relies on fine-tuned balance of protein complexes networks; moreover, impairments of protein interactomes are often the basis of several diseases [11]. Protein–protein interactions (PPIs) in cell signaling represents key triggering complexes and their impairment is at the basis of many human diseases [12]. Particularly, the identification of active peptides able to inhibit and/or modulate functional protein complexes is a promising strategy to develop potential drugs for the treatment of several disorders [25]. Often, structure-based computational docking methods are employed to analyze the peptide–protein binding sites and to provide the most probable conformation of the peptide in complex with its target protein [26]. Moreover, many other complementary approaches including phage display and synthetic peptides screening [27,28] are used to identify novel peptide inhibitors [29]. However, the most part of inhibitors are tested and validated through biochemical assays that are different in procedures and output information, and in accordance to the knowledge of the PPI, different binding assays can be attempted.

On the other hand, several natural peptides with anti- or pro-inflammatory activity have been discovered, in particular, many antimicrobial peptides display anti-inflammatory features [30], they kill Gram negative and/or positive bacteria, *Mycobacterium tuberculosis*, fungi, and cancerous cells [31,32]. In most cases, they are small polycationic peptides able to interact with anionic bacterial surfaces, and able to insert into membrane bilayers, thus forming pores. Alternately, they penetrate into the cell and bind cytoplasmic components crucial to cell living or interfere with cellular metabolism.

Once selected, these active peptides can be optimized for their binding affinity and for their proteases stability by the introduction of non-natural amino acids to generate peptidomimetics. In the case of extracellular targets, peptide inhibitors have direct access, while intracellular uptake of peptides can be achieved by coupling to carrier systems like liposomes or nanoparticles or upon fusion to a protein transduction domain. In the following paragraphs, we focus on recent studies related to protein fragments endowed with anti-inflammatory abilities and perspectives on future applications as therapeutics.

### 2.1. Cancer-Inflammation Diseases

#### 2.1.1. SOCS1/SOCS3 Peptidomimetics

Suppressor of Cytokine Signaling proteins (SOCS) are negative-feedback regulators of the Janus kinase (JAK)/signal transducer of activation (STAT) pathway by reducing the phosphorylation of JAKs and STATs, the consequent dimerization of STATs, and their import into the nucleus, limiting the expression of pro-inflammatory genes [33]. The SOCS family is constituted by eight members (SOCS1–7 and CIS, Cytokine Induced SH2-containing) endowed with a modular structure shared by all proteins; in addition, two of them, SOCS1 and SOCS3, exclusively contain a small domain named kinase-inhibitory region (KIR) that participates in the inhibition mechanism of JAKs activities [34,35]. SOCS1 directly inhibits the catalytic activity of JAK1, JAK2, and Tyrosine kinase 2 (TYK2), but not of JAK3, while SOCS3 is recruited by cytokine receptors that contain high-affinity SOCS3 binding sites (such as glycoprotein (gp)130, LeptinR, granulocyte colony-stimulating factor receptor (GCSFR), etc.). Once attached to these receptors, it can bind to JAK2 via an adjacent surface, forming a ternary complex [36]. SOCS are thought to regulate over 30 cytokines; in detail, SOCS1 controls, preferentially, the expression of interferon- γ (IFNγ) of interleukins (IL)-12/23, 4/13 [37]; instead, the constitutive expression of SOCS3 is able to inhibit lipopolysaccharide (LPS)-induced expression of nitric oxide (NO), tumor necrosis factor- α (TNF-α), and IL-6 [36].

The aberration of the JAK-STAT pathway is often associated with pathologies such as cancers, immune disorders, inflammation, and cardiovascular diseases [38].

The deficiency of SOCS1 protein causes a neonatal fatal inflammatory disease [39], while its overexpression in experimental autoimmune encephalitis (EAE)attenuates IFN-γ destructive effects [40]. Further, the over-expression of SOCS3 inhibits triple negative breast cancer (TNBC) growth and the formation of metastasis in mouse xenograft models, and its loss implies risk of relapses in TNBC patients [41].

In this perspective, the anti-inflammatory functions of SOCS proteins were unveiled and deepened [42,43], and the identification of small compounds able to mimic these SOCS proteins have been carried out by us and other groups [13,14,15,44,45,46,47,48] and are described in following sections.

##### SOCS1 Mimetics

The first SOCS1 mimetic peptide, named Tkip (Table 1), was developed through an in silico complementary approach starting from the sequence of the autophosphorylation site of human JAK2 protein, the fragment corresponding to ^1001^LPQDKEYYKVKEP^1013^ [13]. This approach is used to identify peptide–peptide interactions starting from an antisense RNA that encodes for a hydropathically complementary peptide to the sense RNA [49,50]. Dose-response experiments demonstrated that Tkip blocks altered the autophosphorylation of JAK2 and the phosphorylation of both IFN-γ receptor subunit IFN-gamma receptor (IFNGR)-1 and STAT1 α and reduced the level of pro-inflammatory cytokines like TNFα, as already demonstrated for the entire SOCS1 [13,46].

Also, the activity of the peptide covering the kinase inhibitor region, the KIR peptide of SOCS1, (Table 1), was investigated [13,14,44,45,51]. In vitro studies demonstrated that SOCS1-KIR peptide is able to bind to the JAK2 catalytic site and to inhibit STAT1α phosphorylation. In experimental allergic encephalomyelitis (EAE) mice model, KIR peptide was also able to contrast the action of CD4+ Th1 and Th17 cells on the blood-brain barrier (BBB). Indeed, its presence restored the pathological brain to a physiological state, contrasting the infiltration by Th17 cells [39]. KIR-SOCS1 effects in a type I diabetes mouse model revealed an atheroprotective role for this sequence because it caused improvements of renal functionality and fibrosis and the decrease of pro-inflammatory cytokines such as TNFα and INFγ or C-C motif chemokine ligand (CCL) 25 [52,53].

With the aim to enhance the affinity of KIR sequence toward JAK2 and its stability to proteases’ degradation, we screened a focused peptide combinatorial library in simplified [54] and positional scanning format [55], assuming KIR-SOCS1 as lead compound. The hot-spots within KIR region responsible for the interaction with JAK2 were investigated by performing an Ala-scan on the KIR sequence; this approach revealed that the binding to JAK2 catalytic site is unaltered if alanines are present in positions 54–56, His-Phe-Arg (HFR) and 62–67, Ser- Asp -Tyr-Arg-Arg-Ile, (SDYRRI). On this basis, we performed a deletion-combinatorial screening and identified a new and more potent SOCS1 peptidomimetics, named PS5 (Table 1); in competitive as well as direct binding experiments, PS5 revealed an ability to bind JAK2 catalytic domain with a K_D_ in a low micromolar range (7 µM). Its cellular effects in keratinocytes and in vascular smooth muscle cells (VSMCs) caused a reduction of the phosphorylation levels of STAT1 induced by IFNγ and, similarly, of the STAT1-dependent gene IRF-1 (Interferon-Regulatory Factor) [56].

Furthermore, it inhibited the phosphorylation of STAT3 in response to IL-22, the expression of intercellular adhesion molecule (ICAM)-1 and HLA-DR, the release of CXC motif chemokine ligand (CXCL) 10 and CCL2 by keratinocytes treated with INFγ, and migration of Th1 cells. An ex vivo assay on explants of human skin activated by INFγ confirmed that the treatment with PS5 contrasted INFγ-associated signaling [48].

More recently, starting from the PS5 sequence, we designed several linear and cyclic analogues of PS5, reported in Table 1. Particularly, the phenylalanine in position 58, crucial for the interaction with JAK2 [15], was substituted with a non-natural analogue: naphthylalanine. The serum stability confirmed that cyclic peptidomimetics were stable to proteases’ degradation and the presence of the naphthyl group further increases this stability. Ongoing cellular experiments in mouse VSMCs demonstrated that pretreatment of cells with new analogues reduced STAT1 and STAT3 phosphorylation induced by a combination of pro-inflammatory cytokines (IFNγ plus IL-6). Internalized peptides were also able to inhibit STAT1 nuclear translocation [16].

##### SOCS3 Mimetics

It has been demonstrated that the loss of SOCS3 expression is connected with the onset of basalbreast carcinomas [41]. In the related study, the molecular changes induced by the inactivation of tumor suppressors as phosphatase and tensin homolog (PTEN) and tumor protein 53(p53) were knocked down in normal human mammary cells (HNMECs) and into immortalized homo sapiens mammary gland; breast (MCF10A) ones.

MCF10A/p53^−^/PTEN^−^ cells displayed that the activation of the Stat3/NF-kB pathway induces inflammatory cytokine production, in particular, that of IL6 and transforming growth factor beta (TGF-β), which appeared significantly increased in knocked down cells compared with parental, while the expression of SOCS3 is decreased or undetectable, as well as reduced, in luminal and HER2-positive breast cancer cell lines, or other breast cancer cells that are related to the IL-6 feedback loop [57].

The overexpression of SOCS3 or interfering with IL6 pathway, through the blockade of its receptor, caused the inhibition of the tumor growth and metastasis in mice xenograft models [41]. Similar results are observed in solid tumors, and in malignant fibrous histiocytoma, colorectal, and ovarian cancer cell lines [58]. Thus, targeting IL6/STAT3/SOCS3 axis could be clinically relevant in tumors treatment and in this direction, the development of SOCS3 mimetics could provide an alternative therapeutic approach to interfere with the JAK-STAT pathway in multiple solid cancers. Recently, we analyzed the role of the KIR and ESS (Extended SH2 Subdomain) domains of SOCS3 in the interaction with JAK2 [17]. Relying on X-ray structures of SOCS3-JAK2-gp130 complex [59], we designed and characterized, in vitro, a series of peptides covering KIR and ESS regions mainly involved in JAK2 recognition. Direct binding assays through surface plasmon resonance (SPR) experiments, revealed that both regions contained hot-spots of interactions and that the polypeptide simultaneously covering these protein fragments, named KIRESS, was endowed with the grater affinity toward JAK2, as demonstrated by low micromolar value of its K_D_ reported in comparison with shorter protein fragments in Table 2.

In keratinocytes, KIRESS peptide was able to inhibit the IL-22 molecular signaling by the regulation of STAT3 and extracellular signal-regulated kinase (ERK) 1/2 cascade, as well as the expression of STAT3. In vivo studies revealed that KIRESS peptide reduced tumor growth and activated STAT3 levels in athymic nude mice bearing squamous cell carcinoma (SCC) xenografts [47]. Moreover, very recently, KIRESS peptide was also employed in 4T1 murine and MDA-MB-231, estrogen-negative human, breast cancer cell lines and in vivo mouse xenograft models of the human TNBC subtype. This peptide significantly reduces tumor growth and pulmonary metastasis, proving to be a promising scaffold in TNBC treatment, which currently lacks effective therapies [17].

#### 2.1.2. Aminopeptidase N-term Inhibition in Cancer

**Figure 2:**
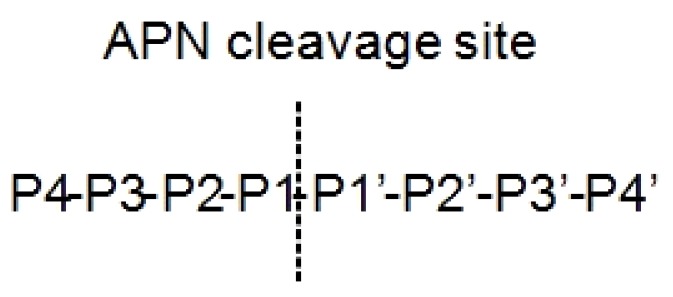
Nomenclature of the aminopeptidase N (APN) peptide substrates. The substrate is cleaved between the P1–P1’.

Aminopeptidase enzymes determine the elimination of amino acids from the N terminus of proteins and/or peptides. They result involved in mechanisms at the basis of different human diseases like cancer and diabetes [60]. Depending on the activities and structures, they are divided in subfamilies: (i) aminopeptidases in a strict sense, (ii) dipeptidyl peptidases, and (iii) tripeptidyl peptidases. A well-studied family is the M1 aminopeptidase, constituted by zinc-dependent enzymes, which regulate cell growth and development [60]. One of the most important members of this family is aminopeptidase N (APN), also called CD13. APN cleaves N-terminal neutral amino acids at P1 substrate position (Figure 2) [61]. It is a dimeric 110kDa protein localized on cell surface constituted by (i) an N-terminal β-domain, (ii) a catalytic transmembrane domain, (iii) a central β-sheet region, and (iv) a C-terminal α-helical domain [62].

APN was over-expressed in many tumors, such as breast, colon, gastric, ovarian, thyroid, and prostate cancers [63], and a selective APN inhibitor as a potential anti-cancer drug, a cyclic peptide named cyc-LHSPW, was identified [18]. A multiplex substrate profiling by mass spectrometry (MSP-MS) approach was employed; a recombinant human APN (rhAPN) was incubated with a library of 228 peptides with 14 residues able to cover an extensive chemical variety to find out active peptides [18].

rhAPN specificity of cleavage resulted for hydrophobic residues in position P1, like norleucine or leucine; on the contrary, aspartic acid and proline negatively interfere with the cleavage. In P4’ position, a positive score was obtained for triptophan, phenylalanine, and proline, and in the P2’ and P3’ positions for serine/threonine and phenylalanine, respectively. In particular, by the MS-MS identification of peptide fragments deriving from only one cleavage, several 5-mer peptides, encompassing P1–P4′ residues, were tested for their inhibition activity, demonstrating the ability to inhibit further cleavage of substrates by rhAPN (Table 3).

Only the peptide NorHSPW (Nor: norleucine) demonstrated the ability to inhibit the cleavage of the substrate, showing an IC_50_ (Median Inhibition Concentration) of 6.5 μM even if the substitution of norleucine with alanine or leucine did not affect the IC_50_ value so much (9.4 μM and 10.6 μM, respectively) (Table 3). In order to rigidify the sequence and reduce the proteolytic degradation, cyclic analogues containing disulfide bridge were developed. The cyclic peptide cyc-LHSPW showed a Ki of 24.7 μM (Table 3).

The inhibitory activity of cyc-LHSPW was tested against several cancer-associated proteases (chymotrypsin, trypsin, aminopeptidase A). APN was the only protease inhibited by cyc-LHSPW with a K_i_ of 24.7 μM, contrary to the others that showed a K_i_ above 100 μM. In vivo experiments on transplant PC3 and DU145 xenografts mice bearing neuroendocrine prostate cancer confirmed that cyc-LHSPW was able to prevent tumor growth in PC3 animals; significantly, contrary to DU145 ones. These data demonstrated that cyc-LHSPW could be a new potent and selective agent active in prostate cancer and showed that MSP-MS technique is a valid approach to identify new APN targeting therapeutics.

### 2.2. Inflammatory Bowel Diseases (IBD)

#### 2.2.1. Chromofungin (CHR: CHGA47–66)

IBD is a chronic illness of the gastrointestinal area that includes ulcerative colitis (UC) and Crohn’s disease (CD). Actual therapeutic approaches for IBD, which involved 5-aminosalicylates, corticosteroids, antibiotics, and immunosuppressive agents, are not satisfactory because of either side effects and lack of efficacy [64]. Patients affected by UC and CD showed, in the mucosa, a severe increase of pro-inflammatory macrophages (M1) [65] able to induce the expression of the nuclear transcription factor kappa B (NF-κB) expression and of enormous amounts of inflammatory mediators such as TNF-α, monocyte chemoattractant protein-1 (MCP-1), and IL-6 [66].

**Figure 3:**
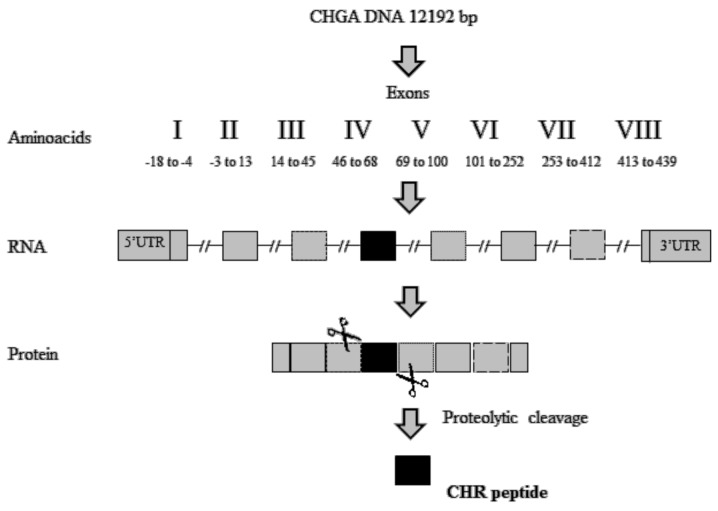
Schematic representations of Chromofungin (CHR) peptide derived from proteolytic cleavage of Human chromogranin-A (CHGA) protein, UTR: UnTranslated Region.

The identification of active peptides able to suppress the activity of macrophages appears to be a valid strategy to contrast these inflammatory disorders. A short peptide derived from proteolytic cleavages of exon-IV of Human chromogranin-A (CHGA) (Figure 3), named Chromofungin (CHR: CHGA47-66) (Table 1), was less expressed in patients with active UC. CHGA is a component of the granins, a group of acidic proteins. Its post-translational modifications, due to a proteolytic cleavage by pro-hormone convertases, led to the release of CHR and other bioactive peptides such as vasostatin, catestatin, pancreastatin, and serpinin [67,68].

In vitro studies revealed that CHR was able to suppress pro-inflammatory macrophage function through the inhibition of toll-like receptor (TLR) 4/NF-κB signaling. Particularly, the blocking of TLR 4/NF-κB pathway induced the reduction of several cytokine expression such as IL-6, IL-1β, TNFα, MCP1, and MIP1 [19].

Moreover, in dextran sulfate sodium (DSS)-induced colitis, the expression of CHR was reduced and, in vivo studies in colitis mice models confirmed that therapy with CHR peptide limited pro-inflammatory cytokines production [68]. Recently, it was pointed out that CHR increased the activity of alternatively activated macrophages (AAMs) in preclinical models [19].

CHR reduced IL-8 levels and improved the expression of arginases, IL-10, Ym1, and TGFβ1 [69], able to promote the repair of tight junction (TJ) that is compromised in IBD and to restore the IECs (intestinal epithelial cells) homeostasis, although further studies are required to confirm its role in intestinal permeability and apoptosis [19].

#### 2.2.2. MC-12 Derived Peptides

NF-κB (nuclear factor kappa-light-chain-enhancer of activated B cells) is a protein complex that controls transcription of DNA and plays a key role in inflammation; normally, it localizes in the cytoplasm, but under inflammatory conditions, it translocates into the nucleus where it regulates the pro-inflammatory gene expression [70].

**Figure 4:**
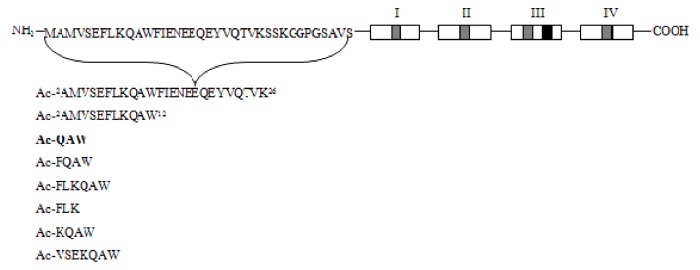
Schematic representation of Annexin protein and derived peptides.

Recent studies revealed a link between NF-κB and anti-inflammatory activity of glucocorticoids as these are able to induce the annexin A1 (ANXA1) expression that, once activated, binds to the p65 subunit of NF-κB, contrasting its activation [71,72]. ANXA1 is a calcium-dependent phospholipid-binding protein [72] composed of two domains: (i) a C-terminal region constituted by four to eight repeats of 70–75 amino acids involved in phospholipid and calcium binding; (ii) a unique N-terminal domain with peptidase, glycosylation, and phosphorylation activity (Figure 4) [73].

It was shown that N-terminal domain of ANXA1 is endowed with the anti-inflammatory activity of the whole protein [74].Thus, two N-terminal derived peptides (Ac2–26 and Ac2–12) were investigated; they demonstrated to be able to reduce NF-κB activity in BxPC-3 (human primary pancreatic adenocarcinoma) and SW480 (human colon adenocarcinoma) cells. With the aim to identify the minimum active sequence of the N-term domain, starting from the Ac2–26 sequence, different short peptides, reported in Figure 4, were investigated. Their action was tested in SW480 cells; only three of six peptides inhibited NF-κB activity, but the most active compound was the tripeptide named MC-12 (Ac-QAW-COOH) including fragment 10–12 (Figure 4) [20,75].

This peptide resulted able to suppress NF-κB activation and confirmed its potential efficacy in colitis using two mouse models of IBD, stimulated with DSS and TNBS (2,4,6- trinitrobenzene sulfonic acid); MC-12 inverted, in a dose-dependent manner, the inflammatory reaction of the colon and prevented ulcerations without apparent side effects in treated animals [76]. However, MC-12 showed that low stability and high doses were required to have effective in vivo responses; further, it was more effective when injected orally [76]. To improve the stability of MC-12 peptide, a backbone cyclization and grafting into cyclic peptide scaffolds, such as SFTI-1 (sunflower trypsin inhibitor 1), was employed. This modification implies the introduction of a bicyclic scaffold [76].

Mono-cyclic versions of SFTI-1 grafted-MC-12 sequences were designed to observe the influence of the cyclic backbone and loop grafted on MC-12 structure and activity. Related sequences are reported in Table 4.

The insertion of the tripeptide MC-12 into the SFTI-1 scaffold improved its therapeutic efficiency. Mainly, in TNBS-induced murine colitis, cyc-MC-12 displayed significantly reduced weight loss after three days, while all other peptides tested, including the linear form of the MC-12 peptide, had no significant effect. In addition, linear peptide MC-12 were completely degraded after 8 h; by contrast, cyc-MC-12 was stable in human serum over this experimental time. The mono-cyclic peptides were more stable than linear MC-12, but were degraded to ≈60% of the initial concentration after 3 h of incubation time (Table 4). The lower stability of the mono-cyclic peptides compared with cyc-MC-12 sequence demonstrates that often the disulfide bond alone is not sufficient to give more in vitro stability and that the cyclic backbone is crucial for this purpose [76].

### 2.3. Autoimmune Disease

#### 2.3.1. IL-15 Antagonist Peptide

Interleukin 15 (IL-15) belongs to the family of cytokines and is produced by different cell types such as monocytes/macrophages, bone marrow stromal and dendritic cells [77]. It interacts with IL-15 receptor constituted by a β subunit (IL-2R/15Rβ) shared with the IL-2 receptor, a common γ subunit and a typical α subunit (IL-15Rα) that confers the receptor selectively to IL-15 [78]. Its expression is upregulated under inflammatory conditions and is associated with a large number of autoimmune disorders like celiac disease, systemic lupus erythematous, multiple sclerosis (MS), and rheumatoid arthritis (RA) [79]. Thus, the identification of IL-15 antagonist peptides could be a potent approach for the treatment of these pathological conditions. A screening of fragments of IL-15, through spot synthesis technique, was carried out to identify crucial residues of IL-15 taking part in the binding with IL-15Rα. This screening implied the use of 22 sequences of 10-mer, encompassing the entire IL-15 sequence, in competitive assays with IL-15R α [80]. Only the increment of signal related to the spot 8, corresponding to the sequence ^36^KVTAMKCFLL^45^ of IL-15, was observed and the related peptide, named P8, was tested through ELISA (Enzyme-Linked Immunosorbent Assay). It demonstrated the ability to contrast the binding of IL-15R to IL-15 in a dose dependent manner, and in CTLL-2 (murine cell line) proliferation assay, an IC_50_ of 130 μM [21]. P8 peptide, containing a free Cys, was able to spontaneously form dimers and its dimer showed an IC_50_ of 24 μM. To investigate the importance of each amino acid in the IL-15Rα binding, an alanine scanning on P8 sequence was performed (Table 5) and peptides were tested.

Phe and Cys appeared to be crucial residues for binding to IL-15Rα, while the substitution of Lysines (36 and 41) with Alanine drastically affected the solubility. Thus, Lys^41^ was substituted with two polar amino acids, glutamic acid and threonine, containing a charged and an uncharged side chain, in peptides named [K6E]P8 and [K6T]P8, respectively. [K6T]P8 peptide showed, in CTLL-2 cells proliferation assays, higher antagonist activity with respect to P8, while [K6E]P8 was inactive and the dimer of [K6T]P8 showed the highest inhibitory activity, in CTLL-2 proliferation assays [81].

The activity of monomer [K6T]P8 was also tested in synovial fluids from rheumatoid arthritis patients that normally showed high levels of IL-15, and consequently of TNFα [82].Both P8 and [K6T]P8 peptides were able to inhibit TNFα secretion, with the latter endowed with greater efficacy [79]. Very recently, [K6T]P8, labeled with technetium-99m (99mTc), showed metabolic stability in synovial fluids from RA patients, and its bio-distribution pattern in healthy rats suggested a slow renal and hepatic elimination. Nevertheless, labeled peptide showed lower solubility compared with P8 peptide [21], and its solubility was tested in three different solvent systems: aqueous buffer added with citric acid, sucrose, and Tween 80. The results indicated that all solvents were able to increase the solubility, but only the sucrose did not affect its biological activity. Considering that sucrose is an excipient used in a large number of peptide-based pharmaceutical formulation, [K6T]P8 could be considered as a potential drug for RA treatment [21].

#### 2.3.2. Cyclotide [T20K]kalata B1 in Multiple Sclerosis

MS is one of the most important autoimmune diseases. Here, the myelin coating on the nerve in the central nervous system (CNS) is damaged and, consequently, the transmission of nerve signals is compromised. In this scenario, the properties of several cyclotides, head-to-tail cyclized peptides with three disulfide bonds, were tested. Particularly, the activity of natural cyclotide kB1 isolated from *Oldenlandiaaffinis* (Rubiaceae) was analyzed. Previous studies showed three distinct sub-regions of kalata B1 molecule: (i) a hydrophilic region (bioactive site); (ii) a hydrophobic face; and (iii) an amendable face (“able to be modified to produce an improvement”) constituted by residues Gly-1, Gly-18, Thr-20, Ser-22, Thr-27, and Asn-29. In order to study the role of each residue of the native sequence of kalata B1, alanine and lysine scanning were performed. Several lysine mutants were not able to inhibit larval development, while others (substitutions in Gly-18, Thr-20, Ser-22, Thr-27, Asn-29, and Gly-1) increased the anthelmintic activity, whereas none of the alanine mutants were observed to be more potent than the wild-type peptide [83,84]. Several mutated peptides covering the hydrophilic region ([G8K], [V10K], and [V10A]) and the amendable face ([G18K], [T20K], and [N29K]) were synthetized and tested. Mutants of the hydrophilic region lost their immunosuppressive activity in carboxy fluorescein succinimidyl ester (CFSE)-labeled lymphocytes or purified T-cells (Table 6), while mutations in amendable face did not affect the anti-proliferative activity.

Based on the IC_50_ values, [T20K] was selected as the most active anti-proliferative peptidomimetic. Its immunological properties were confirmed in its ability to suppress T-cell multi functionality and to block immune-competent cells proliferation. [T20K] peptide reduced the expression of the surface receptor of interleukin-2 (IL-2) and its secretion and gene expression, and production and degranulation activity IFN-γ and TNF-α [22].

In a recent paper, [T20K] mutant was tested in in vivo studies using MS mouse as EAE; through an oral treatment, it significantly reduced progression of the disease and did not show side effects. Thanks to its stability and oral bioavailability, [T20K] could be a promising drug to contrast the progression of MS disease [85].

### 2.4. Neurological Diseases

#### 2.4.1. Neural Cell Adhesion Molecule (NCAM)-Derived Mimetic Peptide and Demyelinating Neurological Diseases

Age-related disorders are connected with changes in the hippocampus region leading to an unbalance secretion of pro- and anti-inflammatory cytokine or decrease of neuronal cluster of differentiation 200 (CD200) expression followed by microglial cell activation. The CD200 expression on neuronal cells is regulated by the anti-inflammatory cytokine IL-4, in fact, IL-4 ^-/-^mice display reduced CD200 expression, which increases when they are treated with exogenous IL-4 [86,87]. Recent studies have demonstrated that a peptide called FGL (fibroblast growth loop) is able to modulate the hippocampus inflammation, to regulate the activation of microglial cells both in vivo and in vitro [23]. FGL is a 15 mer fragment of neural cell adhesion molecule (NCAM) protein spanning residues Glu^681^–Ala^695^; it is involved in the interaction between NCAM and fibroblast growth factor receptor (FGFR). In detail, only its dendrimeric version, four copies of the fragment connected to a three-lysine tree, revealed the ability to bind FGFR1 in SPR experiments [88]. FGL was also tested in a dimeric form, linking two monomers through their N-termini using the iminodiacetic acid [89]. This dimer was able to bind several molecules of FGFR simultaneously producing a realistic peptide/receptor interaction. Particularly, as NCAM, the interaction of FGL with FGFR promotes receptor dimerization with subsequent autophosphorylation of tyrosine residues along its cytoplasmic C-terminal long tail [90].

In addition, FGL induces the secretion of IL-4 from microglial cells in vitro, which leads to the increase of CD200 through ERK signaling, inducing ERK phosphorylation in the hippocampus that inverted the age-related decline [91]. FGL peptide presents several advantages that could make it a promising cognitive enhancer in humans [92]: (i)to cross the blood–brain barrier, (ii) different ways of administration such as directly into the brain ventricles, subcutaneously, or intranasal (iii) enhances social memory in rodents in a dose-dependent manner [89,91]. Human tests are encouraging, but the FGL commercial use is hampered by the administration mode as intravenous bolus.

#### 2.4.2. Microglial Healing Peptide 1(MHP1) in Ischemic Stroke

Ischemic stroke is a result of an occlusion of one or more blood vessels that lead to an instantaneous loss of oxygen from the cerebral tissue, which activates microglial cells that, in turn, induce the secretion of a large number of inflammatory cytokines (TNFα, IL-1β, IL-6) [93].

The inhibition of microglial cells is a valid strategy to reduce the inflammatory process resulting from an ischemic stroke episode [94]. Recently, a direct link between microglial cells activation and the receptor activator of nuclear factor κB/RANK-ligand (RANKL/RANK) pathway was found. Relevant activation of this pathway was able to contrast the activation of microglia cells through TLR, reducing ischemic damage [95]. Even if the identification of enhancers of RANKL/RANK signaling seems to be a great opportunity to develop drugs in ischemic stroke, the principal obstacle is that this pathway is linked to the activation of osteoclast differentiation that causes several bone diseases such as rheumatoid arthritis and osteoporosis.

In order to study the regions of RANKL involved in RANK activation, the crystal structure of mouse RANKL bound to RANK was determined [96]. Binding sites between RANKL and RANK, were reported to be as follows: AA″, CD, DE, and EF loops [96]. Mutagenesis experiments using RANKL mutants (single or multiple substitutions) showed that the AA′′ or AA′′/CD loops are involved in RANK signal-induced osteoclast differentiation. On the contrary, RANKL mutants including DE and EF loops did not induce osteoclast differentiation, but suppressed TLR-mediated inflammation [97].

**Figure 5:**
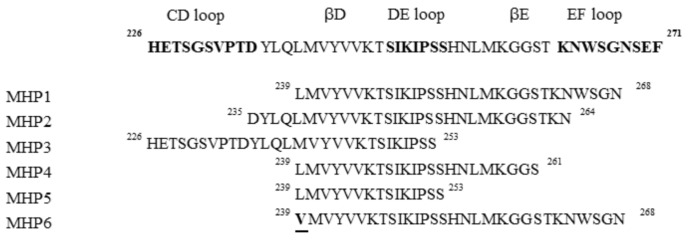
Sequences of microglial healing peptides.

From these results, several peptides deriving from DE and/or EF loops, named microglial healing peptides (MHP), were designed and tested (Figure 5) [24].

Their anti-inflammatory activities were tested on TLR4 using the microglial cell line (MG6). MHP1 and MHP2 demonstrated the ability to inhibit the production of LPS-induced cytokines such as IL-6 and TNF-α, even if MHP1 was the most active. In contrast, the MHP3 peptide, which includes CD and DE loops, showed no inhibitory effects.

With the aim to investigate the biological role of N- and C-termini of MHP1, three peptides, MHP4, MHP5, and MHP6, were designed (Figure 5). These sequences, truncated in the MHP1 C-terminus region, demonstrated to be less effective with respect to MHP1, while MHP6, bearing a single point mutation in 239 position (L → V), completely lost the anti-inflammatory activity. These results indicated the pivotal role of N-terminus in the MHP1 activity. Cellular experiments on RAW264.7 (macrophage cell line) confirmed the anti-inflammatory and anti-osteoclast properties of MHP1, indicating that it could be a valid therapeutic drug in ischemic stroke, and is also able to contrast osteoporosis that is linked to poor prognosis in ischemic stroke [24].

## 3. Conclusions

Nowadays, much attention is focused on peptides able to mimic the function of mediators involved in inflammation-related diseases. Although some of them exist freely in their natural source, the most part of them is encrypted in the architecture of related proteins and can be released by digestion processes or designed on structural bases. Several proteins and peptides from egg, milk, soy, and plant, as well as from marine sources, demonstrated anti-inflammatory properties [98].

In this review, we discuss different examples of peptides that demonstrated anti-inflammatory bioactivity. We reported on several peptide-based mimetics of SOCSs active in both neonatal fatal inflammatory disease and autoimmune encephalitis, as well as in inflammation-cancer processes, just as a small inhibitor of Aminopeptidase N-term in neuroendocrine prostate cancer.

A Chromofungin-derived peptide was capable of suppressing pro-inflammatory action of macrophages by interfering with TLR4/NF-kB signaling and a bicyclic peptide NF-κB activation. Moreover, active peptides in autoimmune diseases such as [K6T]P8, a peptide able to inhibit the receptor of IL-15, and Cyclotide [T20K]kalata B1, an active peptide in multiple sclerosis able to block immune-competent cells proliferation, were described.

Among peptides active in neurological diseases, a fragment of NCAM protein, named FGL peptide, induced the secretion of IL-4 from microglial cells leading to the increase of CD200 and to the inversion of the age related decline of hippocampus in vivo. Further, the MHP1 peptide showed an anti-osteoclast activity in ischemic stroke, by suppressing LPS and TNF-α production. Based on brief examples reported here, we believe that the future growth of peptide drugs will continue, but chemical optimization to improve their action is mandatory. Many different strategies are actually available and can be combined to modify peptides to obtain better therapeutics. One strategy relies on the introduction of conformational constraints, such as mono- or bi-cyclic structures, in order to decrease the conformational flexibility of linear peptides. These modifications often determine an enhancement of membranes permeability and an increase of stability to proteolysis by endo and exopeptidases. Another approach implies the substitution of natural amino acid residues with unnatural ones and/or with N-methyl-α-amino acid that also increases plasma stability of the lead compound [99]. Further budding peptide technologies, including multifunctional peptides, cell penetrating peptides, and peptide drug conjugates, will facilitate the applicability of peptides as therapeutics in the treatment of inflammation disorders, and even beyond.

## Figures and Tables

**Table 1:** Peptide sequences discussed in this review; their molecular activities and the diseases they are involved in are also reported. NF-κB—nuclear transcription factor kappa B; IL—interleukin; LPS—lipopolysaccharide; APN—aminopeptidase N; JAK—Janus kinase; STAT—signal transducer of activation.

Name	Sequences	Activity	Diseases	References
Tkip	WLVFFVIFYFFR	Inhibition of JAK-STAT pathway	inflammatory disease, Autoimmune encephalitis	[13]
SOCS1-KIR	DTHFRTFRSHSDYRRI			[14]
PS-5	DTC(Acm)RQTFRSH		Type-1 skin, cardiovascular diseases	[15]
Cyclic PS5	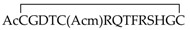			[16]
Linear PS5 Nal1	AcDTC(Acm)RQTNalRSH			[16]
Cyclic PS5 Nal1	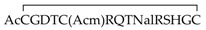			[16]
KIRESS	LKTFSSKSEYQLVVNAVRKLQESG		Triple.negativ-e breast cancer	[17]
cyc-LHSPW	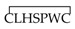	Inhibition of APN	Neuroendocrine prostate cancer	[18]
Chromofungin (CHR: CHGA47-66)	RILSILRHQNLLKELQDLAL	Regulation of alternatively activated macrophages	Inflammatory bowel disease (UC)	[19]
Bi-cyc-MC-12	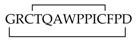	Inhibition of NF-κB expression	Inflammatory bowel disease	[20]
[K6T]P8 peptide	KVTAMTCFLL	Inhibition of IL-15R	Rheumatoid Arthritis	[21]
Cyclotide [T20K]kalata B1	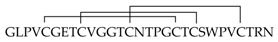	Inhibition lymphocyte proliferation	Multiple sclerosis	[22]
FGL	EVYVVAENQQGKSKA	Stimulate the secretion of IL-4	Demyelinating Neurological Diseases	[23]
MHP1	LMVYVVKTSIKIPSSHNLMKGGSTKNWSGN	Inhibition of LPS-induced cytokine	Ischemical stroke	[24]

**Table 2:** Dissociation constants values (K_D_) of SOCS3 derived peptides toward Janus kinase (JAK2) catalytic domain obtained by surface plasmon resonance (SPR) experiments. KIR—kinase-inhibitory region.

Name	K_D (_μM)
KIR	2.03
ESS	>>1000
KIRESS	1.86

**Table 3:** IC_50_ or K_i_ values of inhibitors of Aminopeptidase N.

Sequence	IC_50_ (μM)	K_i_ (μM)
NorHSPW	6.5	-
AHSPW	9.4	-
LHSPW	10.6	-
Cyc-LHSPW	-	24.7

**Table 4:** Sequences of the grafted MC-12 peptides. The native MC-12 sequence is underlined.

Name	Sequence	% Peptide Remaining in Serum
SFTI-1	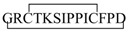	≈ 100
Bi-cyc-MC-12	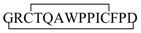	≈ 100
Mono-cyc-MC-12 (n)	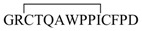	≈ 60
Mono-cyc -MC-12 (p)	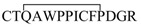	≈ 60
Mono-cyc -MC-12 (l2)	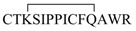	≈ 60

**Table 5:** Peptide sequences deriving from IL-15 36–45 fragment, P8, obtained by point mutations. IC_50_ values were obtained in proliferation assay, ND: Not Detected.

Name	Sequence	IC_50_ (µM)
P8	KVTAMKCFLL	130
P8 dimer	KVTAMKCFLLdimer	24
[K1A]P8	AVTAMKCFLL	ND
[V2A]P8	KATAMKCFLL	130
[T3A]P8	KVAAMKCFLL	130
[M5A]P8	KVTAAKCFLL	130
[K6A]P8	KVTAMACFLL	ND
[C7A]P8	KVTAMKAFLL	inactive
[F8A]P8	KVTAMKCALL	inactive
[L9A]P8	KVTAMKCFAL	200
[L10A]P8	KVTAMKCFLA	260
[C7S]P8	KVTAMKSFLL	inactive
[K6E]P8	KVTAMECFLL	inactive
[K6T]P8	KVTAMTCFLL	24.6
[K6T]P8 dimer	KVTAMTCFLLdimer	8.0

**Table 6:** IC_50_ of kalata B1 and derived peptides in lymphocytes and purified T-cells, PBMC: Peripheral Blood Mononuclear Cell.

Peptide	IC_50_ (µM) Lymphocytes (PBMCs)	IC_50_ (µM) Purified T-cells
native kalata B1	2.9	2.4
[T8K]	Inactive	-
[V10A]	Inactive	-
[V10K]	Inactive	-
[G18K]	4.4	3.2
[T20K]	1.9	2.7
[N29K]	3.2	2.1
